# Prognostic and molecular multi-platform analysis of CALGB 40603 (Alliance) and public triple-negative breast cancer datasets

**DOI:** 10.1038/s41523-025-00740-z

**Published:** 2025-03-08

**Authors:** Brooke M. Felsheim, Aranzazu Fernandez-Martinez, Cheng Fan, Adam D. Pfefferle, Michele C. Hayward, Katherine A. Hoadley, Naim U. Rashid, Sara M. Tolaney, George Somlo, Lisa A. Carey, William M. Sikov, Charles M. Perou

**Affiliations:** 1 Bioinformatics and Computational Biology Curriculum, University of North Carolina at Chapel Hill, Chapel Hill, NC USA; 2 Lineberger Comprehensive Cancer Center, University of North Carolina at Chapel Hill, Chapel Hill, NC USA; 3 Department of Genetics, University of North Carolina, Chapel Hill, NC USA; 4 Department of Biostatistics, University of North Carolina, Chapel Hill, NC USA; 5 Dana-Farber/Harvard Cancer Center, Boston, MA USA; 6 City of Hope Comprehensive Cancer Center, Duarte, CA USA; 7 Division of Hematology-Oncology, Department of Medicine, School of Medicine, University of North Carolina, Chapel Hill, NC USA; 8 Program in Women’s Oncology, Women and Infants Hospital of Rhode Island, Warren Alpert Medical School of Brown University, Providence, RI USA

**Keywords:** Breast cancer, Cancer genomics, Breast cancer

## Abstract

Triple-negative breast cancer (TNBC) is an aggressive and heterogeneous disease that remains challenging to target with traditional therapies and to predict risk. We provide a comprehensive characterization of 238 stage II-III TNBC tumors with paired RNA and DNA sequencing data from the CALGB 40603 (Alliance) clinical trial, along with 448 stage II-III TNBC tumors with paired RNA and DNA data from three additional datasets. We identify DNA mutations associated with RNA-based subtypes, specific *TP53* missense mutations compatible with potential neoantigen activity, and a consistently highly altered copy number landscape. We train exploratory multi-modal elastic net models of TNBC patient overall survival to determine the added impact of DNA-based features to RNA and clinical features. We find that mutations and copy number show little to no prognostic value, while RNA expression features, including signatures of T cell and B cell activity, along with stage, improve stratification of TNBC survival risk.

## Introduction

Triple-negative breast cancer (TNBC), characterized by the lack of estrogen receptor (ER), progesterone receptor (PR), and human epidermal growth factor receptor-2 (HER2) overexpression or gene amplification, represents approximately 15% of all breast tumors^1^. In early-stage TNBC, the addition of carboplatin and an immune checkpoint inhibitor (ICI) to neoadjuvant chemotherapy has recently demonstrated a significant increase in the pathologic complete response (pCR) rate and a reduction in the risk of recurrence. However, patients with residual disease after four chemotherapies plus ICI still have a three-year event-free survival of only 67%^2^. Thus, additional chemotherapy and other drugs are given with ICI adjuvantly in this high-risk group of patients^2–4^. While several molecular predictors of breast cancer have been developed^5–8^, only clinical factors like pre-treatment tumor size and node status, and the presence or absence of residual disease, are currently used to tailor the treatment in early-stage TNBC (exception: germline BRCA mutation status for PARP inhibitor treatment). For this reason, the development of accurate TNBC prognostic tools to guide escalation and de-escalation strategies is a key unmet need.

From a molecular perspective, TNBC tumors are largely basal-like (60-80%) by PAM50 gene expression subtyping^9^, and they have the highest mutation rates and copy number alteration frequencies when compared to other breast cancer subtypes^10^. Notably, the tumor suppressor gene *TP53* is somatically mutated in approximately 80% of all TNBC tumors^10^. Many of these are missense mutations that produce mutant p53 proteins, making them attractive as potential TNBC therapeutic targets^11,12^. TNBC also exhibits a unique tumor microenvironment, being the most immune-activated breast cancer subtype by tumor-infiltrating lymphocytes (TILs) levels, expression of the programmed death-ligand 1 (PDL1) protein, and immune-gene expression signatures^13^. What is also important is that these differences in tumor microenvironment have clinical implications. TNBC patients with higher TILs and B cell/T cell gene expression have significantly higher pCR rates to neoadjuvant chemotherapy with and without ICI, as well as better survival outcomes^14–17^.

CALGB 40603 is a phase II clinical trial (now part of the Alliance for Clinical Trials in Oncology) with a 2 × 2 factorial design that enrolled participants with stage II-III TNBC and tested the addition of carboplatin or bevacizumab to neoadjuvant weekly paclitaxel followed by dose-dense doxorubicin and cyclophosphamide. While the addition of either carboplatin or bevacizumab was found to significantly increase pathologic complete response, the primary endpoint of the study^18^, the addition of either carboplatin or bevacizumab was not found to significantly increase event-free survival, the secondary endpoint of the study^15^. Importantly, both DNA-sequencing and RNA-sequencing was performed on pre-treatment samples from this study, providing a valuable dataset resource of stage II-III TNBC samples with paired DNA and RNA data. While the CALGB 40603 RNA-sequencing data was initially presented along with the study’s secondary endpoint results^15^, we newly report here the CALGB 40603 DNA-sequencing data in this study.

To expand our understanding of the molecular and prognostic landscape of TNBC, we present the DNA sequencing data from the CALGB 40603 randomized phase II clinical trial as part of an integrated, multi-omic characterization of matched DNA and RNA sequencing data across 686 stage II-III TNBC patients from four datasets. Using this data, we train and evaluate exploratory prognostic models of overall survival to gain insights into how molecular features may be valuable in furthering TNBC precision medicine efforts.

## Results

### Baseline patient characteristics

The primary dataset of interest is Cancer and Leukemia Group B (CALGB) 40603, for which we are newly presenting the pre-treatment targeted panel DNA-seq data from 238 stage II-III TNBC patients. All 238 patients are TNBC following current guidelines and have matched pre-treatment DNA-seq tumor, DNA-seq blood, and RNA-seq tumor samples (Supplementary Fig. 1)^15^. To validate our findings and increase our sample size, we compiled paired DNA and RNA data from stage II-III TNBC patients from three additional publicly available datasets: Fudan University Shanghai Cancer Center (FUSCC, *n* = 224)^19^, the Molecular Taxonomy of Breast Cancer International Consortium (METABRIC, *n* = 91)^20^, and The Cancer Genome Atlas (TCGA, *n* = 133)^10^. In total, 686 stage II-III TNBC patients are included in the study and are summarized in Table 1. The median age of all patients is 51 years (IQR, 43–59) and the percentage of stage II patients in each dataset ranges from 69.7% to 81.3%. In all four datasets, most samples are PAM50 basal-like (56% to 81.9%), but the PAM50 intrinsic subtype proportions differ significantly across the four datasets (*p* < 0.001).

**Table 1:** Baseline characteristics of TNBC patients by dataset

Characteristic	Patients, No. (%)					*P*-value
	CALGB 40603	FUSCC	METABRIC	TCGA	All	
	(*n* = 238)	(*n* = 224)	(*n* = 91)	(*n* = 133)	(*n* = 686)	
Median age, years (IQR)	48 (40–56)	53 (45–60)	50 (41–59)	55 (47–62)	51 (43–59)	
Clinical stage						
II	166 (69.7)	180 (80.4)	74 (81.3)	104 (78.2)	524 (76.4)	0.026
III	72 (30.3)	44 (19.6)	17 (18.7)	29 (21.8)	162 (23.6)	
PAM50 molecular subtype						
Basal-like	195 (81.9)	153 (68.3)	51 (56.0)	105 (78.9)	504 (73.5)	< 0.001
Claudin low	4 (1.7)	11 (4.9)	27 (29.7)	3 (2.3)	45 (6.6)	
HER2-enriched	16 (6.7)	52 (23.2)	9 (9.9)	11 (8.3)	88 (12.8)	
Luminal A	11 (4.6)	1 (0.4)	3 (3.3)	10 (7.5)	25 (3.6)	
Luminal B	0 (0)	4 (1.8)	1 (1.1)	2 (1.5)	7 (1.0)	
Normal-like	12 (5.0)	3 (1.3)	0 (0)	2 (1.5)	17 (2.5)	
Overall survival						
Median follow-up, years	7.6 (6.5–8.1)	7.3 (6.2–8.9)	18.3 (12.3–21.3)	2.6 (1.2–5.8)	7.3 (5.7–8.5)	
Somatic mutation data	238 (100)	167 (74.6)	90 (98.9)	133 (100)	628 (91.5)	
Copy number data	238 (100.0)	215 (96.0)	91 (100)	133 (100)	677 (98.7)	

In addition to DNA and RNA data, each dataset has long-term survival data. For this study, we focused on overall survival, where an event represents death from any cause. The unadjusted overall survival proportions of each dataset over time are shown in Supplementary Fig. 2. The four datasets have significantly different baseline survival trajectories (*p* < 0.001), with the FUSCC dataset having the highest overall survival rate and the METABRIC dataset having the lowest overall survival rate. METABRIC is also the oldest dataset with the longest median follow-up time of 18.3 years (IQR: 12.3–21.3 years) (Table 1). TCGA has the shortest median follow-up time of 2.6 years (IQR: 1.2–5.8 years), and the median follow-up time of all combined samples is 7.3 years (IQR: 5.7–8.5 years). As expected, tumor stage is a significant predictor of overall survival across all four datasets (Supplementary Fig. 3), where higher stage (III vs II) corresponds to worse survival.

### Mutational landscape of CALGB 40603

**Fig. 1:**
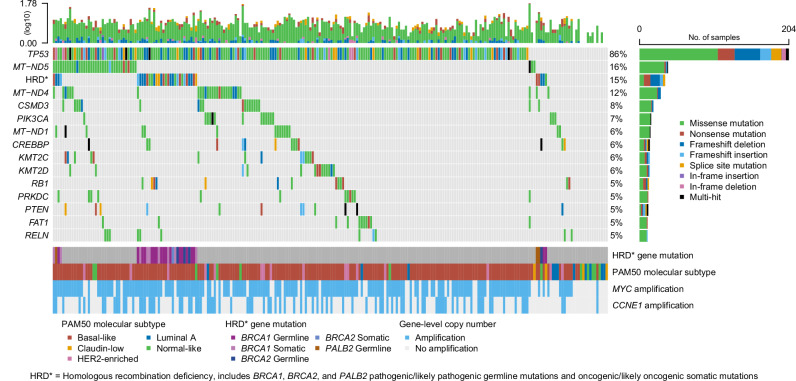
The mutational landscape of the CALGB 40603 dataset. The columns correspond to individual patients (*n* = 238) and the rows correspond to mutations of the 14 genes with the highest somatic mutation frequencies and a homologous recombination deficiency (HRD) feature, representing any *BRCA1*, *BRCA2*, or *PALB2* pathogenic/likely pathogenic germline mutation or oncogenic/likely oncogenic somatic mutation. Color-coded labels correspond to mutation type, with light gray representing wildtype. Patient-level and gene-level mutation frequency distributions are shown at the top and right, respectively. RNA-based (PAM50 subtype) and DNA-based (*MYC* and *CCNE1* amplification) annotations for each patient, including annotations for the HRD gene mutations, are included at the bottom with corresponding legends.

Somatic variant calling on the CALGB 40603 paired tumor-normal DNA-seq data identified a total of 2093 mutations (1861 single nucleotide variants, 188 deletions, and 44 insertions) across the 1037 genes included in the targeted panel. A visualization of the most frequent mutations (excluding known passenger hotspot mutated genes) in the CALGB 40603 dataset is shown in Fig. 1. Notably, *TP53* mutations were present in 204 samples (86%), followed by low (<20%) mutation rates for every other gene. Other notable mutated cancer-related genes include *PIK3CA* (7%), *CREBBP* (6%), *KMT2C* (6%), *KMT2D* (6%), *RB1* (5%), *PTEN* (5%), and *FAT1* (5%), all of which are reported as “Tier 1” cancer-related gene mutations in the COSMIC Cancer Gene Census^21^. Interestingly, three mitochondrial genes, *MT-ND5* (16%), *MT-ND4* (12%), and *MT-ND1* (6%), were among the most frequently mutated in the dataset.

Additionally, pathogenic/likely pathogenic germline and oncogenic/likely oncogenic somatic mutation calls were made for the *BRCA1*, *BRCA2*, and *PALB2* genes and were considered indicators of homologous recombination deficiency (HRD). In total, there were 35 CALGB 40603 samples with HRD mutations, including 27 samples with *BRCA1* mutations, 5 samples with *BRCA2* mutations, and 3 samples with *PALB2* mutations (Supplementary Fig. 4). If these HRD mutations are considered as a single feature alongside somatically mutated genes, the mutation frequency (15%) would be the third most frequent in the CALGB 40603 set (Fig. 1); *MYC* and *CCNE1* were frequently amplified (75.2% and 37.4%, respectively), predominately in the samples with *TP53* mutations.

Differences in mutation frequencies across PAM50 RNA-based subtypes were tested using univariate binomial generalized linear models. The basal-like subtype (*n* = 195) was used as the reference subtype for the comparison of mutation frequencies versus the HER2-enriched (*n* = 16) and Luminal A (*n* = 11) subtypes. In total, there were six statistically significant mutation differences by subtype (Table 2). Four genes had a significantly higher mutation frequency in HER2-enriched vs. basal-like samples: *PIK3CA* (FDR-adj *p* < 0.001), *PTEN* (FDR-adj *p* = 0.0043), *PIK3R1* (FDR-adj *p* = 0.0059), and *NF1* (FDR-adj *p* = 0.027). *PIK3CA* was also significantly higher in luminal A than in basal-like (FDR-adj *p* = 0.025), and *TP53* was significantly lower in luminal A than in basal-like (FDR-adj *p* < 0.001).

**Table 2:** Mutations that are significantly different between PAM50 subtype comparisons (FDR-adj *p* < 0.05)

Reference subtype	Alternative subtype	Gene mutation	FDR-adj p-value	Proportion of mutated basal-like subtype	Proportion of mutated alternative subtype
Basal-like	HER2-enriched	*PIK3CA*	< 0.001	6/195 (3%)	7/16 (44%)
Basal-like	HER2-enriched	*PTEN*	0.0043	6/195 (3%)	5/16 (31%)
Basal-like	HER2-enriched	*PIK3R1*	0.0059	3/195 (2%)	4/16 (25%)
Basal-like	HER2-enriched	*NF1*	0.027	2/195 (1%)	3/16 (19%)
Basal-like	Luminal A	*TP53*	< 0.001	181/195 (93%)	3/11 (27%)
Basal-like	Luminal A	*PIK3CA*	0.025	6/195 (3%)	3/11 (27%)

We next tested the association of somatic mutations with response and survival. Using univariate binomial generalized linear models, we tested for differences in mutation frequencies according to pathologic complete response status. Comparisons of residual disease vs. pCR for all CALGB 40603 patients, patients receiving bevacizumab, and patients receiving carboplatin were tested, but no gene was significant after multiple test corrections. To find associations with mutation status and overall survival in the CALGB 40603 set, we fit univariate Cox proportional hazards models and found that mutated *PIK3R2* (2.5% frequency) was associated with worse overall survival, with FDR-adj *p* < 0.001, HR = 11.98, 95% CI (4.91-29.20). No other mutation was significant after multiple test corrections. Additionally, we tested the pathogenic/likely pathogenic germline and oncogenic/likely oncogenic somatic mutation calls for the *BRCA1*, *BRCA2*, and *PALB2* genes in CALGB 40603. No combination of germline/somatic mutation in these three genes (individually or combined) were associated with pCR or overall survival for all CALGB 40603 patients and for only patients receiving carboplatin.

### Mutational landscape across TNBC datasets

We next looked at somatic mutations across all four datasets combined, as this greatly increased our sample size and power (*n* = 628). A visualization of the most frequent mutations across all combined TNBC samples is shown in Supplementary Fig. 5. *TP53* was again the most frequently mutated (84%), and *PIK3CA* frequency rose to 14%. The cancer-associated genes *PTEN* (7%), *KMT2C* (7%), *KMT2D* (7%), *RB1* (6%), and *CREBBP* (4%), were again among the 15 most frequently mutated and at similar frequencies as observed in CALGB 40603. We did not have the germline data to make the HRD mutation calls for the other three datasets, but somatic *BRCA1* mutations alone comprised 3% of samples. Other “Tier 1” cancer-related gene mutations in the COSMIC Cancer Gene Census^21^ present in the 15 most frequently mutated genes include *PIK3R1* (4%), *NF1* (4%), *NOTCH1* (4%), *APC* (3%), and *ATR* (3%).

**Fig. 2:**
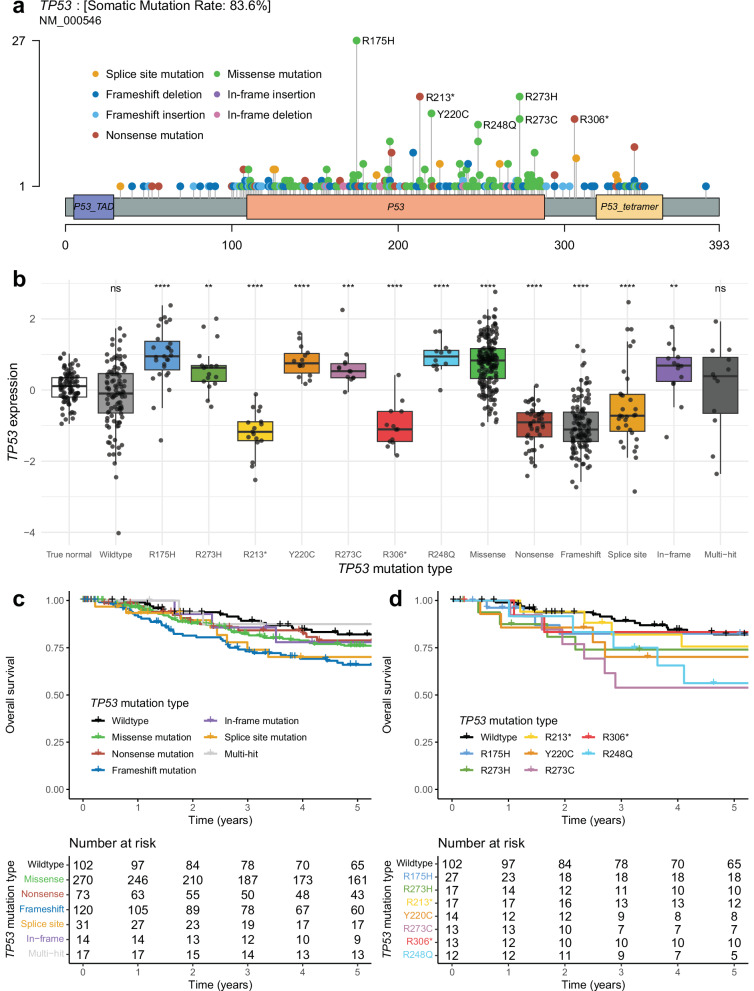
Somatic *TP53* mutations among samples from four combined datasets (CALGB 40603, FUSCC, METABRIC, and TCGA). **a** Lollipop plot showing the distribution of *TP53* mutations among patients. The x-axis depicts *TP53* amino acid location, and amino acid mutations are depicted as lollipops at the location where they occur, with the color corresponding to the mutation type and height corresponding to the number of patients with that specific mutation. **b** *TP53* normalized RNA expression by *TP53* mutation type, including cancer-adjacent normal samples from TCGA. Asterisks represent significant Wilcoxon rank sum tests comparing the expression of samples with each *TP53* mutation type to the *TP53* expression of the normal samples, adjusted for multiple tests (*FDR-adj *p* ≤ 0.05, **FDR-adj *p* ≤ 0.01, ***FDR-adj *p* ≤ 0.001, ****p ≤ 0.0001). **c** Kaplan–Meier plot depicting the overall survival proportion of patients over time by their *TP53* mutation type. **d** Kaplan–Meier plot depicting the overall survival proportion of patients over time by the status of recurrent *TP53* mutations and *TP53* wildtype.

In the combined cohort, we identified 538 *TP53* mutations across 525 samples. This gave a large enough sample size to look at recurrent *TP53* mutations, with frequencies shown in Fig. 2a. There were seven *TP53* mutations present in at least 10 patients: R175H (*n* = 27), R273H (*n* = 17), R213* (*n* = 17), Y220C (*n* = 14), R273C (*n* = 13), R306* (*n* = 13), and R248Q (*n* = 12). *TP53* mRNA expression was significantly higher in samples with missense and in-frame mutations, while samples with nonsense, frameshift, and splice site mutations had significantly lower *TP53* expression than cancer-adjacent normal breast tissue samples (Fig. 2b). We compared the overall survival of samples with each *TP53* mutation classification (missense, nonsense, frameshift, in-frame, splice site) to the overall survival of wildtype *TP53* and found that samples with frameshift mutations had significantly worse overall survival than samples with wildtype *TP53*, with *p* = 0.034, HR = 1.74, 95% CI (1.04-2.89) (Fig. 2c). We did similar overall survival comparisons of the seven recurrent *TP53* mutations vs. wildtype *TP53* and found samples with the R273C *TP53* mutation had significantly worse overall survival than wildtype *TP53*, with *p* = 0.015, HR = 2.88, 95% CI (1.22–6.79) and samples with the R248Q *TP53* mutation had significantly worse overall survival than wildtype *TP53*, with *p* = 0.034, HR = 2.57, 95% CI (1.08–6.16) (Fig. 2d).

**Fig. 3:**
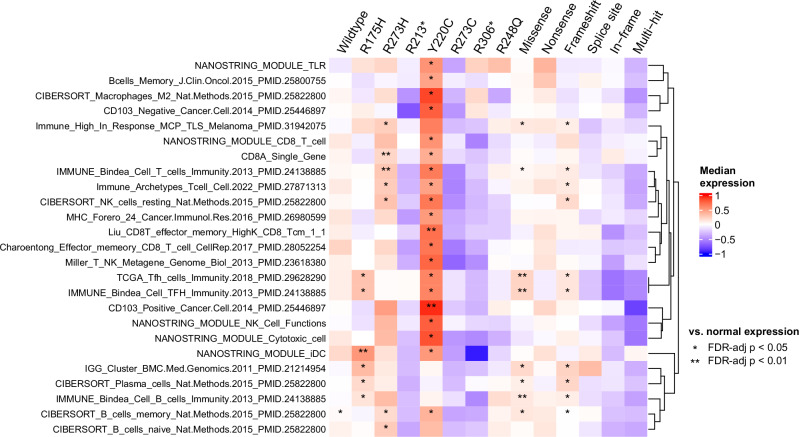
Immune gene signatures (rows) by *TP53* mutation type (columns), with each cell representing the expression of the corresponding signature in samples with the corresponding *TP53* mutation type. Annotations represent the significance of a one-sided Wilcoxon rank-sum test comparing the immune signature expression of samples with the corresponding *TP53* mutation type vs. the immune signature expression of normal samples, adjusted for multiple tests (*FDR-adj *p* ≤ 0.05, **FDR-adj *p* ≤ 0.01). The immune signatures shown in the heatmap represent those with FDR-adj *p* < 0.05 for at least one recurrent *TP53* missense mutation and FDR-adj *p* ≥ 0.05 for *TP53* nonsense mutations. Rows are hierarchically clustered.

Recent work has shown that some recurrent *TP53* missense mutations may act as neoantigens, with evidence of immunogenicity and recognition by human T cells^22,23^. Given this, we looked at the expression of 233 immune signatures (Supplementary Data 2) to see if there were any that had significantly high expression in samples with any of the six recurrent (*n* ≥ 10) *TP53* missense mutations from the combined TNBC samples (R175H, R273H, Y220C, R273C, R248Q) compared to expression in normal samples (Wilcoxon FDR-adj *p* < 0.05). As a control, we selected only signatures without significantly high expression in samples with nonsense *TP53* mutations (unlikely neoantigens) compared to expression in normal samples (Wilcoxon FDR-adj *p* ≥ 0.05); 25 total immune signatures fit these criteria, many of which were signatures of adaptive immunity (Fig. 3). Of the six recurrent *TP53* missense mutations, three (R175H, R273H, Y220C) had significantly higher expression in at least six of these immune signatures than in normal samples (Wilcoxon FDR-adj *p* < 0.05).

### Copy number alteration patterns within and across TNBC datasets

**Fig. 4:**
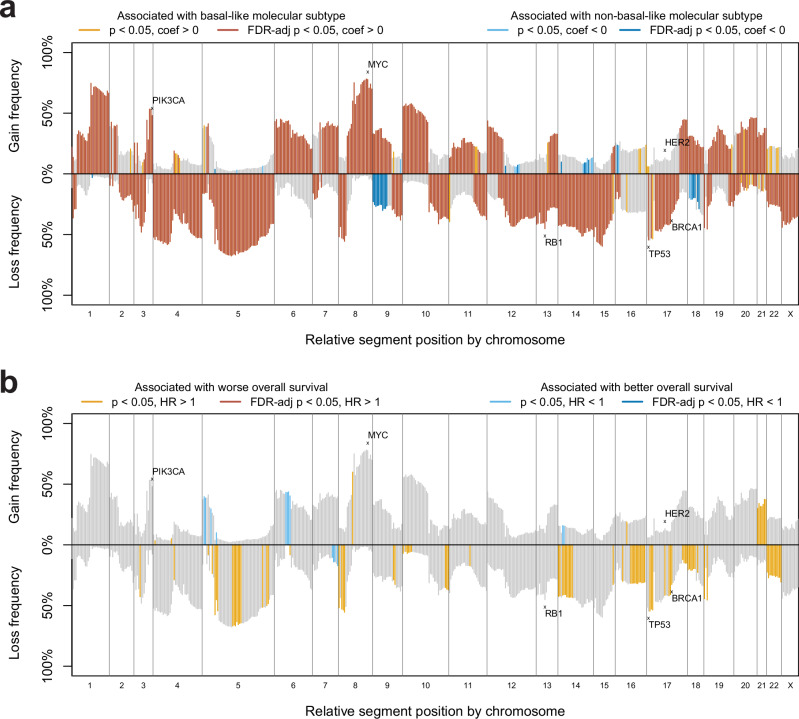
Segment-level copy number landscape plots of the combined TNBC samples. On the x-axis, each of the 534 copy number segments is plotted in relative order, with height above the x-axis corresponding to the gain frequency of the segment within the sample set and height below the x-axis corresponding to the loss frequency of the segment within the sample set. **a** segment gain/loss frequencies are colored by statistical significance and direction of association of binomial generalized linear models using segment gain/loss status to predict basal-like subtype. Orange-colored segment gains/losses are statistically more significant in basal-like samples vs. non-basal-like samples with (dark orange) and without (light orange) multiple test corrections. Blue-colored segment gains/losses are statistically more significant in non-basal-like samples vs. basal-like samples with (dark blue) and without (light blue) multiple test corrections. **b** segment gain/loss frequencies are colored by statistical significance and direction of association of Cox proportional hazards models using segment gain/loss status to predict overall survival. Orange-colored segment gains/losses are associated with worse survival, with (dark orange) and without (light orange) multiple test corrections. Blue-colored segment gains/losses are associated with better survival, with (dark blue) and without (light blue) multiple test corrections.

We evaluated DNA copy number alterations in each of the four TNBC datasets at the level of 534 predefined chromosomal segments, including whole-arm segments and regions with demonstrated importance across cancers^24^. Notably, the copy number landscapes of each of the four TNBC datasets were very similar, characterized by the same distinct shape of segment-level gain/loss frequencies when plotted (Supplementary Fig. 6 and Supplementary Fig. 7). In each dataset, the highest gain frequencies come from 8q segments, followed by 1q, 3q, and 10p segments, and the highest loss frequencies come from 5q segments, followed by 4p, 4q, 8p, 15p, and 17p segments. The characteristic regions of high and low copy number alterations for these TNBC datasets closely resemble those of the genomic-based classification IntClust 10 from Dawson et al., which is known to be highly associated with the basal-like molecular subtype^25^. For each dataset, differences in segment-level copy number gains and losses in PAM50 non-basal-like vs. basal-like subtype were tested using univariate binomial generalized linear models (Supplementary Fig. 6, Supplementary Data 1). Many segment gains and losses in each dataset were found to be associated with basal-like subtype after adjusting for multiple test corrections; copy number gains from 1q, 3q, 6p, 9p, and 12p segments had FDR-adj *p* < 0.05 in at least 3 datasets, and copy number losses from 4p, 4q, 5q, 12q, and 14q segments had FDR-adj *p* < 0.05 in all four datasets. We then performed the same analysis after combining the TNBC samples from all four datasets to give a larger sample size (*n* = 677). A total of 225 segment-level copy number gains and 309 segment-level copy number losses were associated with higher frequency in basal-like samples compared to non-basal-like samples (FDR-adj *p* < 0.05), and a total of 12 segment-level copy number gains and 18 segment-level copy number losses were associated with higher frequency in non-basal-like samples compared to basal-like samples (FDR-adj *p* < 0.05) (Fig. 4a, Supplementary Data 1). We next tested associations of segment-level copy number gains and losses with overall survival using univariate Cox proportional hazards models. No segment-level copy number gain or loss was statistically significant in any individual dataset or in the 677 TNBC samples combined after multiple test corrections, though some segments were associated with either better or worse overall survival without FDR adjustment. (Fig. 4b, Supplementary Fig. 7, Supplementary Data 1). For CALGB 40603, we found no segment-level copy number gains and losses in patients statistically associated with residual disease or pCR using univariate binomial generalized linear models after multiple test corrections (Supplementary Fig. 8, Supplementary Data 1).

### Exploratory molecular elastic net models of TNBC overall survival

**Fig. 5:**
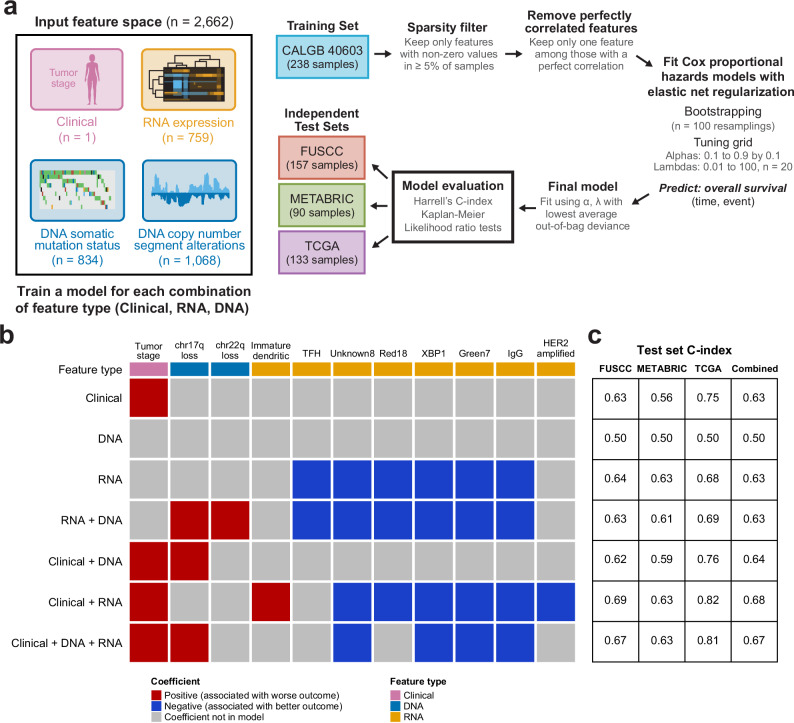
Multi-platform models of overall survival in patients with stage II-III TNBC. **a** Schematic overview of the workflow used to train and evaluate the Cox proportional hazards models with elastic net regularization. This workflow was used to train a model for each combination of input feature type (clinical, RNA, and DNA). Note that the clinical-only model only has one input feature (tumor stage), so this workflow was not used and instead a Cox proportional hazards model was fit to the training set without bootstrapping or regularization. **b** Each model by the coefficients in the final model, colored by positive (red) or negative (blue) coefficient value. **c** The C-index values of each model in the three individual test sets and in the combined test set.

Finally, we wanted to see if we could build prognostic models of TNBC overall survival using DNA-based, RNA-based, and clinical features. Importantly, we wanted to explore if DNA- and RNA-based features showed any prognostic value in early-stage TNBC, both on their own and when considered with tumor stage. To evaluate this, we chose to train Cox proportional hazards models with elastic net regularization, as elastic net models are interpretable and can handle high-dimensional data. Our input feature space consisted of one clinical feature (tumor stage), 759 RNA features (predetermined RNA expression signatures^26^), and 1929 DNA features (the gain/loss status of 534 predetermined DNA copy number segments^24^, the somatic mutation status of 727 genes, and the somatic mutation status of 134 recurrent *TP53* mutations). For each combination of input feature data (clinical, DNA, RNA), we trained a separate Cox proportional hazard model of stage II-III TNBC overall survival on the CALGB 40603 dataset (*n* = 238). Because the clinical-only model had only one input feature, no elastic net regularization was used. In all other models, features were selected via elastic net regularization with a bootstrapping approach used to select the optimal regularization parameters. All models were then evaluated using data from three independent tests FUSCC (*n* = 157), METABRIC (*n* = 90), and TCGA (*n* = 133) (Fig. 5a) that were not used to train the models.

Among the seven models, only eleven total features were selected from the possible combined *n* = 2662 input feature space. These included tumor stage, two DNA features (loss of chr 17q and 22q segments), and eight RNA features (immature dendritic cell, T-follicular helper cell, XBP1, IgG, and HER2 amplification signatures, as well as three unsupervised RNA signatures labeled Unknown8, Red18, and Green7). To better characterize these three unsupervised signatures, we performed gene set enrichment analysis of each signature using the Human MSigDB gene sets; the most enriched gene sets included RAY_TUMORIGENESIS_BY_ERBB2_UP (FDR-adj *p* < 0.001) for Unknown8, GSE11884_WT_VS_FURIN_KO_NAIVE_CD4_TCELL_UP (FDR-adj *p* = 0.003) for Red18, and GSE4142_NAIVE_VS_MEMORY_BCELL_UP (FDR-adj *p* = 0.002) for Green7, suggesting HER2 amplification, T cell, and B cell associations, respectively (Supplementary Data 4)^27^. The tumor stage, chr17q loss, chr22q loss, and immature dendritic features had positive model coefficients and were associated with worse overall survival, while all other features had negative model coefficients and were associated with better overall survival. (Fig. 5b). The coefficient values of the selected features for each model are listed in Supplementary Data 3. Model discrimination was assessed with the Harrell’s C-index metric of each model on the independent test set data, shown in Fig. 5c. All models except for the DNA-only model, which selected no features during training, appeared prognostic, with C-index values greater than 0.6 on the combined test set data. Overall, DNA features did not appear to add much value to any of the models. Even though DNA somatic mutations were included in the input feature space, none of these features were selected by the models, and while two DNA copy number features were selected by the models that considered other feature types in addition to DNA, the C-index values did not appear to be any stronger when compared to the models with the same input feature space but without DNA features. On the other hand, RNA features were prognostic on their own, achieving an equivalent C-index (C = 0.63) in the combined test data to the clinical stage-only model. Additionally, the clinical + RNA model had the highest C-index (C = 0.68).

**Fig. 6:**
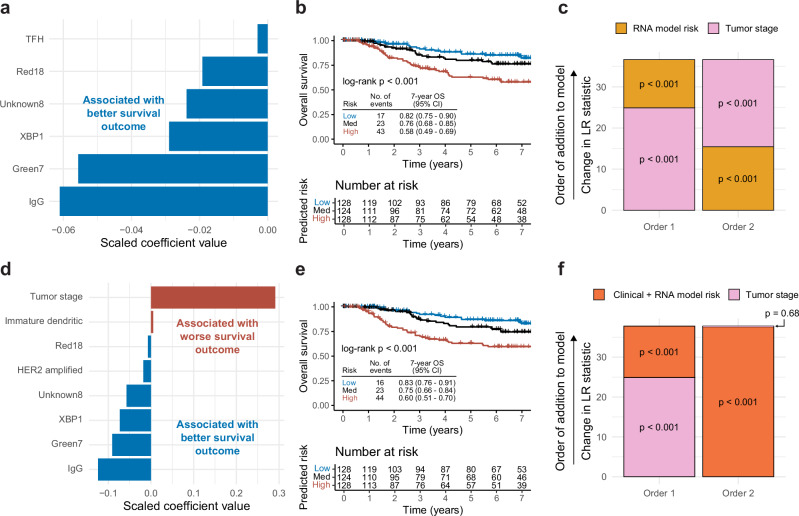
RNA-only and clinical + RNA models of overall survival in patients with stage II-III TNBC. **a**–**c** corresponds to the RNA-only elastic net model, and **d**–**f** corresponds to the clinical + RNA elastic net model. **a**, **d** The features selected by the elastic net model and their corresponding scaled coefficient values. Features with negative values (blue) are associated with better overall survival and features with positive values (red) are associated with worse overall survival. **b**, **e** Kaplan–Meier plots of overall survival by predicted survival risk from the corresponding elastic net model. Continuous risk scores predicted for each sample were categorized into low-risk (blue), medium-risk (black), and high-risk (red) cutoffs based on the median risk score of each test set. Samples and associated risk scores from the three test sets were combined. **c**, **f** The likelihood-ratio (LR) statistic was estimated as we added the continuous elastic net risk score and/or tumor stage to a Cox proportional hazards model using the samples from the combined test set. The change in LR statistic when tumor stage, then risk score is added is shown (order 1) alongside the change in LR statistic when RNA model risk, then tumor stage is added is shown (order 2). The *p*-values displayed represent the statistical significance of the corresponding coefficient in the univariate/multivariate model on test set data.

Because the RNA molecular feature type appeared to have prognostic value in the test data, we chose to highlight the RNA-only model and the clinical + RNA model in more detail (Fig. 6). First, in the RNA-only model, all six selected RNA features had negative coefficients (associated with better overall survival), with the IgG signature having the strongest contribution to the risk score, followed by the Green7 (B cell associated) signature (Fig. 6a). The ability of the RNA-only model risk score to discriminate overall survival in the combined test set samples was visualized with a Kaplan–Meier plot in Fig. 6b, with high/medium/low risk patients separated by tertile (log-rank *p* < 0.001). Corresponding Kaplan–Meier plots of the RNA-only model risk score in each individual test set are included in Supplementary Fig. 9a–c. Furthermore, we wanted to estimate whether the RNA-only model provided independent information from tumor stage (the “clinical standard”); to evaluate this, we incorporated the continuous RNA-only risk score and tumor stage as predictors in Cox proportional hazards models on the combined test data, stratified by set. Notably, the RNA-only risk score coefficient remained significant after the addition of tumor stage (*p* < 0.001), and the likelihood-ratio (LR) statistic increased 47% when the RNA-only risk score was added to tumor stage. In the opposite order, the tumor stage coefficient also remained significant after the addition of the RNA-only risk score (*p* < 0.001) (Fig. 6c). The clinical + RNA model contains eight total features, including tumor stage and five of the same features from the RNA-only model (IgG, Green7, XBP1, Unknown8, and Red18). Tumor stage (associated with worse overall survival) has the strongest contribution to the model, followed by the IgG signature (associated with better overall survival) (Fig. 6d). A Kaplan–Meier plot visualizing the ability of the clinical + RNA model’s ability to discriminate high/medium/low risk patients by tertile in the combined test data (log-rank *p* < 0.001) is included in Fig. 6e, and corresponding individual Kaplan–Meier plots for each individual test set are included in Supplementary Fig. 9d–f. While the clinical + RNA model incorporates tumor stage as a feature, we wanted to assess whether it provided any additional meaningful prognostic information compared to tumor stage alone. As with the RNA-only model, we evaluated this by incorporating the continuous clinical + RNA risk score and tumor stage as separate predictors in univariate and multivariate Cox proportional hazards models of overall survival on the combined test data. The clinical + RNA risk score remained significant after the addition of tumor stage (*p* < 0.001), and the LR statistic increased 52% when the clinical + RNA risk score was added to tumor stage. In the other direction, the tumor stage coefficient did not remain significant (*p* = 0.68) after the addition of the clinical + RNA risk score, which is consistent with the fact that tumor stage was already used to calculate the risk score and should not add any new information (Fig. 6f).

Lastly, because the CALGB 40603 DNA-sequencing data came from a 1037 gene targeted panel, we didn’t have the appropriate coverage to accurately calculate HRD scores based on genomic scar algorithms^28^, but we were interested in if these scores may have added prognostic value to our models. To assess this, we took publicly available HRD scores calculated on two of our test datasets, namely the FUSCC and TCGA cohorts^19,29^ and incorporated the continuous HRD score and the clinical + RNA risk score as separate predictors in univariate and multivariate Cox proportional hazards models of overall survival on the respective FUSCC and TCGA test sets. In the FUSCC test set, HRD score was not significant in a univariate model (*p* = 0.42), and it remained insignificant when it was added to the clinical + RNA risk score in a multivariate model (*p* = 0.22) (Supplementary Fig. 10a). In the TCGA test set, HRD score was significant in a univariate model (*p* = 0.013), and it remained significant when it was added to the clinical + RNA risk score in a multivariate model (*p* = 0.042), although we note that the LR statistic had a very small increase with this addition (0.7%) (Supplementary Fig. 10b).

## Discussion

Treatment of early-stage TNBC remains a challenge in oncology because of limited targeted therapies, low survival rates, and a lack of clinical predictors of survival other than tumor stage and the presence of residual disease after neoadjuvant treatment. The analysis of integrated, multi-omic high-throughput sequencing data provided here from CALGB 40603 provides a greater molecular resolution and is an important community resource of TNBC genomic and clinical data, which has increased our understanding of the disease. In this study, the CALGB 40603 mutational and DNA copy number landscape was largely characterized by features that have previously been reported in other studies, including a high *TP53* mutation rate and widespread genomic instability with frequent 8q gains and 5q losses^10^. Interestingly, multiple mitochondrial genes were among the most frequently mutated in CALGB 40603 (*MT-ND5*, *MT-ND4*, *MT-ND1*). Previous work has identified mutations in these mitochondrial genes, though more research is needed to evaluate the role they may play in TNBC^30^.

Next, gene expression subtype-specific analyses of CALGB 40603 mutations confirmed the enrichment of *TP53* mutations in PAM50 basal-like vs. luminal A tumors, which has been documented^10^. These analyses also highlighted a possible association of the PAM50 HER2-enriched vs. basal-like subtype with the PI3K/AKT pathway, with significantly higher relative mutation frequencies of *PIK3CA*, *PIK3R1*, *PTEN*, and *NF1* in the HER2-enriched subtype when analyzed within those clinically defined as TNBC. *PIK3CA* and *PIK3R1* mutations are both known activators of the PI3K/AKT pathway, while *PTEN* is a known inactivator of the pathway^31^. *NF1* is a known inactivator of RAS signaling, which stimulates the PI3K/AKT pathway^32^. *NF1* loss has also been identified as a resistance mechanism to PI3K^33^. Separately, mutated *PIK3R2*, another known activator of the PI3K/AKT pathway, was found to be associated with worse overall survival across all CALGB 40603 samples, but its overall mutation frequency was low (2.5%).

To the best of our knowledge, this is one of the largest molecular and prognostic analyses of stage II-III TNBC using samples with paired DNA and RNA data, with *n* = 686 total samples across datasets. This large, combined sample size provided increased statistical power for the analysis of recurrent *TP53* mutations. We identified a total of seven recurrent *TP53* mutations found in ten or more patients across the combined data, all of which have been previously reported across multiple cancer types^34^. Compared to normal breast tissue *TP53* mRNA expression, we found that TNBC *TP53* mRNA expression was significantly higher in samples with missense and in-frame mutations and significantly lower in samples with nonsense, frameshift, and splice site mutations. This finding makes sense in the context of nonsense-mediated mRNA decay (NMD), the cellular surveillance pathway that degrades mRNAs containing premature stop codons. Nonsense, frameshift, and splice site *TP53* mutations can all trigger NMD, leading to reduced *TP53* mRNA levels, while missense and in-frame mutations do not typically degrade NMD, which may lead to increased *TP53* mRNA levels. From a survival standpoint, we found samples with *TP53* frameshift mutations, *TP53* R273C, and *TP53* R248Q mutations to have significantly worse overall survival compared to samples with wildtype *TP53*. This is consistent with a recent analysis by Pal et al. that characterized the phenotypes of cancer cell lines expressing common missense p53 mutations and found p53 R273C and R248Q mutants to be among the most aggressive (in addition to R248W and Y220C)^35^. Additionally, we found three recurrent *TP53* missense mutations (R175H, R273H, and Y220C) that showed immune signature expression associations consistent with possible neoantigen activity. This is reinforced by previous findings by Kim et al., which identified human T cell receptors with tumor cell reactivity that target both p53 R175H and Y220C^23^. Concrete steps toward therapeutically targeting p53 R175H and Y220C in patients are already underway. A synthetic bispecific antibody that binds to p53 R175H, activating T cells to kill tumor cells developed by Hsiue et al. was effective at lysing tumor cells both in vitro and in vivo in animal models^36^, and a phase I/II clinical trial (PYNNACLE) evaluating the efficacy of PC14586, a p53 reactivator developed by PMV Pharmaceuticals that is selective for p53 Y220C, alone and in combination with pembrolizumab is currently underway^37^. The potential of *TP53* R273H as a neoantigen is also supported by work from Yuan et al., which demonstrated that *TP53* R273H elicited peptide-specific T cells in vitro^38^.

One of the goals of this study was to evaluate the prognostic value of DNA and RNA features in early-stage TNBC, both alone and when considering tumor stage, which is already used for clinical decision-making. We explored this by training Cox proportional hazards models of overall survival with elastic net regularization for each combination of DNA, RNA, and clinical features. While we did not find strong evidence to support the prognostic value of DNA features, RNA features did appear to be prognostically valuable, both on their own and, more importantly, after tumor stage was considered. The eight RNA expression signatures selected by these elastic net models help provide biological insight into these prognostic associations. Most notably, we see themes of B cell and T cell activity being associated with better overall survival among the selected features. In both the RNA-only and the clinical + RNA elastic net models, the immunoglobulin G (IgG) signature^39^ had the strongest model contribution of all selected RNA features. This is consistent with IgG evenness being one of the most prognostic features of CALGB 40603 event-free survival in Shepherd et al.^15^. IgG is an antibody created and released by released by plasma B cells; another selected unsupervised RNA signature, Green7^26^ showed association with B cell activity through gene set enrichment analysis. Selected RNA features associated with T cell activity include the Red18^26^ signature (via gene set enrichment analysis) and a T follicular helper cell (TFH) signature. This observation of B cell and T cell signatures being associated with better TNBC overall survival is supported by findings from Hollern et al., which found that TFH activation of B cells facilitated anti-tumor response in TNBC murine models^40^. Interestingly, even though these elastic net models were trained entirely on HER2 negative (by IHC/FISH) patients, a HER2 amplified signature^26^ was among the selected RNA features, along with the Unknown8^26^ unsupervised signature, which was associated with *ERBB2* (HER2) tumorigenesis through gene set enrichment analysis. Both signatures were associated with better TNBC overall survival. Among the genes that comprise the RNA signatures selected by these models are *CXCL13*, which encodes a B cell chemoattractant, *CCL19*, which encodes a T cell chemoattractant, *ICOS*, which encodes a T cell costimulator, and *PDCD1*, which encodes the PD-1 cell surface receptor on T cells and B cells and is a cancer immunotherapy target^41^.

There are several important limitations of our study. While we tried to maintain a uniform sample group by selecting stage II-III TNBC patients receiving chemotherapy, there were significant differences in the distribution of important phenotypes between the four datasets analyzed in this study that we couldn’t control for, including tumor stage, PAM50 intrinsic subtype, and baseline overall survival. Given the inherent heterogeneity within TNBC, future efforts that perform similar analyses on more homogeneous TNBC subpopulations may be valuable. While we filtered our patients for those only receiving chemotherapy, we acknowledge that there was uncontrolled variability in the exact treatment regimen across patients and datasets. We also acknowledge that because all patients received some sort of treatment, our efforts to define “prognostic” biomarkers is more technically an effort to define “mixed prognostic/predictive” biomarkers. Additionally, there were differences between sequencing technologies used to quantify the DNA and RNA data used in this analysis. The METABRIC dataset is older than the other three sets, so microarray technology was used to quantify RNA expression instead of RNA-seq. CALGB 40603 and METABRIC mutation calls were made from targeted DNA sequencing data, while FUSCC and TCGA mutation calls were made from whole exome sequencing (WES) data. DNA copy number was quantified from targeted DNA sequencing data for CALGB 40603, SNP array data for METABRIC and FUSCC, and WES data for TCGA. Given the coverage limitations of a 1,037 gene DNA-sequencing panel in detecting genome-wide measurements in the CALGB 40603 dataset, we did not perform mutational signature analyses or compute HRD scores based on genetic scar algorithms. Though we could not include these features in our prognostic model training space, we did attempt to evaluate whether a genome-wide HRD score would add value to our models in the TCGA and FUSCC sets, which have publicly available HRD scores. While the HRD score did not add significant prognostic value to the clinical + RNA model risk score in the FUSCC data, it did add significant prognostic value in TCGA, although it only provided a very small increase to the likelihood ratio statistic (<1%); thus, knowledge of HRD status may not add much additional value beyond the clinical + RNA model for TNBC patients receiving multi-agent chemotherapy. Furthermore, we would have liked to train multivariate elastic net models that predict pCR with clinical, RNA, and DNA features in a similar approach to our survival models. Because CALGB 40603 was the only dataset with pCR information, we were underpowered to train and test such models, though this would be an interesting future analysis with more data.

In conclusion, we performed a comprehensive characterization of the RNA- and DNA-based molecular and prognostic landscape of stage II-III TNBC. Future studies are needed to validate the findings of TNBC patients with recurrent *TP53* missense mutations that show phenotypes consistent with neoantigen activity. While somatic molecular features are not yet clinically evaluated for stage II-III TNBC, we show that RNA-based features, including those of B cell and T cell activity, may add prognostic value to tumor stage when considering overall survival. Thinking to the future, improved prognostic estimates could be important to informing treatment escalation/de-escalation efforts; however, further work and validation are needed before creating a molecular predictor that is appropriate and feasible for clinical use.

## Methods

### CALGB 40603 study design and patient cohort

CALGB 40603 is a 2 × 2 factorial, randomized phase II trial that evaluated the impact of adding carboplatin and/or bevacizumab to standard chemotherapy. The study design and clinical results of CALGB 40603 have previously been published^15,18^. Eligible patients for the trial included stage II-III triple-negative (ER and PR staining ≤10%, HER2 IHC 0-1+ or FISH < 2 if IHC 2+ or no IHC available) invasive breast cancer. Patients received paclitaxel 80 mg/m^2^ once a week for 12 treatments, followed by doxorubicin plus cyclophosphamide once every two weeks for four treatments. Each patient was randomly assigned to receive no additional treatment, the addition of bevacizumab 10 mg/kg once every 3 weeks for 9 treatments, the addition of carboplatin (area under the curve = 6) once every 3 weeks for four treatments, or both. The UNC Office of Human Research Ethics has determined that the correlative science research does not constitute human subjects research as defined under federal regulations (Study #: 18-0846). The CALGB 40603 trial protocol was approved by the central institutional review board of the National Cancer Institute, as well as institutional review boards at the participating sites. All patients enrolled in CALGB 40603 provided informed consent in accordance with federal and institutional guidelines. CALGB is now part of the Alliance for Clinical Trials in Oncology.

### CALGB 40603 targeted DNA sequencing data

Genomic DNA (gDNA) was extracted from tumor tissue frozen in liquid nitrogen and matched blood samples using the Qiagen DNeasy Blood & Tissue Kit, according to the manufacturer’s protocol as previously described^42^. gDNA quantity was assessed using the Invitrogen Qubit dsDNA broad range assay (Q32853) with the Thermo Scientific Qubit 3.0 Fluorometer (Q33216), following the MAN0002325 protocol. gDNA quality was assessed using the TapeStation DNA Genomic ScreenTape analysis (5067-5365) with the Agilent TapeStation 4200 instrument (G2991AA), following the G2991-90040 protocol. Up to 1.5 µg of gDNA was used as input for library preparation with the Agilent SureSelect XT Kit (G9641B) and Agilent Bravo Automated Liquid Handler, following the G7530-90000 protocol. Fragmentation was performed with the Covaris Ultrasonicator Instrument (Model E220) and single, 8 bp indexes were used. The UNCseq^TM^ version 10.0 (Design 3065031) targeted capture panel, manufactured by Agilent (5190-4833) was used. Library quantity was assessed using the Invitrogen Qubit dsDNA high sensitivity assay (Q32854) with the Thermo Scientific Qubit 3.0 Fluorometer (Q33216), following the MAN0002326 protocol. Library quality was assessed using the DNA ScreenTape analysis (5067-5582) with the Agilent TapeStation 4200 instrument (G2991AA), following the G2991-90031 protocol. Sequencing was performed on an Illumina HiSeq4000 instrument with 2 × 75 bp paired-end reads to an average sequencing depth of 30 million clusters per library (~1700X raw sequencing depth).

### Patient inclusion in analysis

To be consistent with the current clinical definition of TNBC, CALGB 40603 samples with ER and PR staining >1% were excluded from analysis along with samples that did not pass DNA quality control metrics. The three external datasets evaluated, FUSCC, METABRIC, and TCGA, were additionally filtered to resemble the CALGB 40603 patient population as closely as possible given the available phenotypic data. For the FUSCC dataset, all samples were TNBC (ER and PR staining ≤1%, HER2-), and were filtered to include only stage II-III samples treated with chemotherapy. For the METABRIC dataset, PR IHC and HER2 FISH data were unavailable, so TNBC was defined as IHC ER- (staining ≤10%) and HER2- (IHC 0-1 + , or HER2 SNP6 Loss/Neutral if IHC 2+ or no IHC available). The METABRIC dataset was filtered to include only stage II-III TNBC samples treated with chemotherapy. Additionally, the TCGA dataset was filtered to include only stage II-III TNBC (ER and PR staining ≤ 10%, HER2 IHC 0-1+ or FISH < 2 if IHC 2 + ).

There was a total of 686 tumor samples with available RNA and DNA data after combining the four datasets, but only a total of 677 samples with available copy number data, 628 samples with somatic mutation data, and 619 samples with both copy number and somatic mutation data. In total, there were 618 samples with RNA expression, DNA copy number, DNA somatic mutation, and overall survival data (all features needed for training/testing the elastic net model). The corresponding largest subset of samples not including NAs was used for each type of analysis. For some combined analyses involving *TP53* expression, 97 TCGA cancer-adjacent normal samples were included.

### Mutation calling

For CALGB 40603 and TCGA samples, paired tumor and normal DNA fastq files were aligned to the hg38 reference genome by BWA mem, with sorting, indexing, and marking of duplicate reads by Biobambam2 bamsormadup^43^. Somatic mutations were called from bam files with Strelka2^44^ and Mutect2^45^. Mutation calls were merged and filtered for only those marked as ‘PASS’ by both variant callers. Calls were annotated and converted to MAF format with Ensembl Variant Effect Predictor (VEP) and the vcf2maf tool^46^.

For FUSCC and METABRIC samples, where paired tumor and normal DNA fastq files were not accessible, already-processed mutation calls were used. FUSCC mutation calls (FUSCCTNBC_Mutations_Extended_hg38.maf) were downloaded from Figshare (10.6084/m9.figshare.19783498.v5)^47^ and METABRIC mutation calls (somaticMutations.txt) were downloaded from GitHub (https://github.com/cclab-brca/mutationalProfiles/tree/master)^48^. All downloaded mutation calls were then re-annotated using Ensembl VEP and the maf2maf tool^46^.

To avoid the downstream analysis of known passenger hotspot mutations, any mutations in the list of 194 genes confidently under neutral selection from Hess et al. were filtered out from each dataset^49^. Additionally, for consistency, only mutations present in the 1037 genes from the UNCSeq v10 targeted panel were considered for any dataset in all analyses.

Gene-level somatic mutation analysis was performed by binarizing somatic mutation calls at the gene level for each patient, where a status of 1 indicates at least one mutation called for a gene in a patient and a status of 0 indicates no mutation calls for a gene in a patient.

For *BRCA1*, *BRCA2*, and *PALB2* in CALGB 40603, filtered somatic mutations were evaluated using the ClinGen/CGC/VICC 2022 guidelines^50^. Mutations labeled oncogenic or likely oncogenic following these guidelines were considered a potential source of homologous recombination deficiency. Additionally, germline *BRCA1*, *BRCA2*, and *PALB2* mutations in CALGB 40603 called by Strelka2 in germline mode were evaluated using the ACMG/AMP 2015 guidelines^51^. Mutations labeled pathogenic or likely pathogenic following these guidelines were considered a potential source of homologous recombination deficiency.

Mutational landscape plots (oncoplots) and lollipop plots were created using the maftools R package (v2.18.0). Mitochondrial genes were removed from the oncoplot of the combined data (Supplementary Fig. 5) because these genes are not included in most whole exome capture panels.

### DNA copy number data

For CALGB 40603 and TCGA samples, paired tumor and normal DNA fastq files were aligned to the hg38 reference genome by BWA mem, with sorting, indexing, and marking of duplicate reads by Biobambam2 bamsormadup^43^. Bam files were used as input for ASCAT (v3.1.2), run with the default workflow for targeted sequencing data for CALGB 40603 and with the default workflow for whole exome sequencing data for TCGA^52^. For FUSCC samples, probe-level OncoScan CNV Assay data were downloaded via the NCBI Gene Expression Omnibus (GSE118527)^19^. 23 of the FUSCC tumor samples had paired white blood cell samples available; these were used as input for ASCAT (v3.1.2), run with the default workflow for SNP array data. The other 401 FUSCC tumor samples that had no paired normal samples available; were run with the ASCAT (v3.1.2) default workflow for SNP array data without matched normal data. For the METABRIC samples, copy number segment files generated via ASCAT were downloaded from GitHub (https://github.com/cclab-brca/mutationalProfiles/tree/master)^48^.

The allele-specific ASCAT outputs were used to produce log2 ratio copy number scores by dividing the total copy number by the tumor ploidy estimate: ${\log }_{2}(\frac{{nAraw}+{nBraw}}{{ploidy}})$. For each dataset, this was used to run GISTIC2 (v2.0.23), with the following parameters changed from the default: –genegistic 1 –broad 1 –brlen 0.5 –conf 0.95 –armpeel 1 –savegene 1 –ta 0.3 –td 0.3 –rx 0^53^. The gene-level GISTIC2 copy number output was then collapsed to 534 segment-level copy number scores, which include whole-arm segments and predefined chromosome regions that have previously been published and shown to be significant in pan-cancer analyses or breast cancer subtype-specific analyses^24,54–58^. Segment-level copy number scores were calculated by taking the mean of the GISTIC2 copy number scores of genes within each segment. The full lists of genes used to determine the copy number scores of the 534 segments are given in Xia et al. (excluding the two Y chromosome segments)^24^. Segment-level copy number scores above 0.3 were considered a copy number gain, and segment-level copy number scores below −0.3 were considered a copy number loss.

### RNA expression data

For CALGB 40603, TCGA, and FUSCC samples, tumor RNA-seq fastq files were aligned to the hg38 reference genome with Gencode v36 annotations^59^ via STAR (v2.7.6a)^60^, and quantification was performed with Salmon (v1.4.0)^61^. Salmon counts were normalized using a fixed upper quartile based on all non-zero transcripts and were subsequently log2 transformed with a pseudocount of one. For METABRIC, normalized Illumina HT 12 microarray RNA expression data was downloaded from the European Genome-phenome Archive at the European Bioinformatics Institute (EGAS00000000083) and were subsequently log2 transformed with a pseudocount of one^20^.

759 published RNA expression signatures representing biological pathways, cell types, disease states, and important single genes were calculated from the normalized RNA expression data of each dataset. These signatures have been previously partially summarized^26^, and a complete list of each signature, its source, and the method used to calculate it (e.g. median expression, correlation to centroids, special algorithm from original methods) is given in Supplementary Data 2. RNA expression signatures were initially calculated from the entire normalized sample set of each initial study before each dataset was subset to include only stage II-II TNBC samples. Once a dataset was filtered to only include samples relevant to the analysis, gene signature expression estimates were median-centered. These are the gene signature expression estimates that were used in downstream analyses.

For a few analyses, *TP53* expression values were combined between datasets (including TCGA adjacent normal samples). To minimize batch effects, *TP53* expression in each dataset was scaled and centered before it was combined.

### Tumor subtyping

PAM50 molecular subtypes were determined using a clinical subgroup-specific gene-centering method based upon Zhao et al.^62^, as previously detailed^63^. For the CALGB 40603 and FUSCC datasets, the TNBC samples from the original PAM50 training set were used to create TNBC subgroup-specific gene centering columns, which were used to individually normalize the expression values of the PAM50 genes in the CALGB 40603/FUSCC sets and apply the PAM50 predictor^64^. For the TCGA and METABRIC datasets, which initially contained samples belonging to all three clinical subtypes, samples were split into HER2+, HR+/HER2−, and TNBC sample groups and the expression values of the PAM50 genes in the TCGA/METABRIC sets were normalized separately based on the respective original PAM50 HER2+, HR+/HER2−, or TNBC training set samples. The normalized expression data of each clinical subtype was then recombined before applying the PAM50 predictor. Claudin-low classifications were made for each dataset after the initial PAM50 calls as previously documented, by cross-referencing a Claudin-low centroid predictor^65^ with hierarchical clustering of the dataset by the intrinsic gene list from Parker et al.^64^; samples that were centroid positive and clustered together were labeled Claudin-low. As with the PAM50 calling, for each dataset, calls were made from the full RNA datasets of each initial study (including samples with RNA but no matching RNA and non-TNBC samples, if any).

### Prognostic model building

We were interested in the ability of three types of input features to predict overall survival in stage II-III TNBC: clinical features (tumor stage), DNA features (700 gene-level somatic variants, 134 *TP53* somatic variants, 534 segment-level copy number gains, and 534 segment-level copy number losses), and RNA features (759 gene expression signatures). Therefore, we trained seven unique models on the CALGB 40603 dataset (*n* = 238), with each model considering each combination of input feature type (clinical, DNA, RNA) (Fig. 5). Because there was only a single input feature for the clinical-only model (tumor stage, representing the current “clinical standard”), we fit a Cox proportional hazards model with no regularization to the entire training set. For the six other models, we fit Cox proportional hazards models with elastic net regularization the same multi-step training workflow. Because sparse features can make feature selection via elastic net less stable, the first step of the training workflow was to remove sparse features, defined as features with non-zero values in ≤5% of training samples. Features were further filtered to keep only one feature among any with perfect correlations in the training set to avoid model instability and non-unique solutions. A bootstrapping approach was then used to fit Cox proportional hazards models with elastic net regularization to the training set. The elastic net regularization is a combination of the lasso (L1) and ridge (L2) regularization penalties, and it is incorporated to prevent the Cox proportional hazards model from overfitting when incorporating many model coefficients^66^. A total of *n* = 100 bootstrapped resamplings were fit to a tuning grid of nine alphas (0.1–0.9 by 0.1) and twenty lambdas (10^2^ to 10^−2^, evenly spaced on a logarithmic scale), using the glmnet R package (v4.1-8). The alpha/lambda combination with the lowest average out-of-bag deviance was then chosen to fit a final model on all training set samples. All seven models were then evaluated using three independent test sets: FUSCC (*n* = 157), METABRIC (*n* = 90), and TCGA (*n* = 133). For the models that used feature selection, the model coefficients in their original scale were extracted from the fit glmnet model, and the corresponding scaled coefficient values following the default standardization method used by glmnet were manually re-calculated so the relative contribution of each selected feature to the model could be evaluated. Additionally, Harrell’s C-index values were used to assess model discrimination, along with Kaplan–Meier plots split by tertile-based risk scores (survminer R package v0.4.9). Because tumor stage is the feature representing the “clinical standard”, we also wanted to test if the models incorporating molecular features could discriminate overall survival risk beyond the predictions made from tumor stage. To evaluate this, for the elastic net models incorporating molecular features, we performed likelihood ratio tests, using the elastic net model risk score and tumor stage as predictors in univariate and multivariate Cox proportional hazards models on the test data. In the test data, models were first conditioned on tumor stage, and then the significance of the elastic net model was tested (the same was done in the opposite order).

Gene set enrichment analysis was performed on three unsupervised RNA expression signatures (Green7, Red18, Unknown8) that were selected by the elastic net models, using the enricher function from the clusterProfiler R package with all human gene sets from MSigDB, with the Benjamini & Hochberg method use for *p*-value adjustment^27,67,68^. The enrichment results with adjusted *p*-values < 0.05 for each unsupervised signature is included in Supplementary Data 4.

### Statistical analyses

All statistical analyses were performed in R (v4.3.3). Comparisons of differences in baseline phenotypic variables in Table 1 were made using Chi-squared tests. Kaplan–Meier plots were created using the R package survminer (v0.4.9), with default log-rank *p*-values displayed. All multiple test correction was done using the p.adjust function in R with the Benjamini & Hochberg method to control the False Discovery Rate (FDR)^68^. The significance threshold of *p* < 0.05 was used for all analyses unless otherwise specified.

The Wilcoxon rank sum test was used to calculate the statistical significance when comparing continuous expression values across sample sets. Two-sided Wilcoxon rank sum tests were run with multiple test correction testing unequal *TP53* expression of samples with *TP53* mutation types vs. normal samples (Fig. 2b). In analyses relating to Fig. 3, one-sided Wilcoxon rank sum tests were run with multiple test correction testing greater expression of 233 immune signatures for samples with *TP53* mutation types vs. normal samples. Immune signatures that had an FDR-adj Wilcoxon *p* < 0.05 in samples with a recurrent (*n* ≥ 10) missense *TP53* mutation and an FDR-adj Wilcoxon *p* ≥ 0.05 in samples with a *TP53* nonsense mutation were subjected to complete-linkage hierarchical clustering with Euclidean distance.

For all univariate survival analyses, overall survival associations were modeled by Cox proportional hazards models: Overall survival (time, event) ~feature, using the survival package in R (v3.7-0). For survival analyses that combined samples across datasets, the model was stratified by set: Overall survival (time, event) ~feature + strata(set). pCR and subtype associations were modeled with binomial generalized linear models: pCR status ~feature and subtype ~feature. For subtype analyses that combined samples across datasets, the dataset was added as a fixed effect to the model: subtype ~feature + set.

Mutation subtype, pCR, and survival analyses considered any gene-level mutation or *TP53* mutation present in five or more CALGB 40603 samples, as well as HRD mutation status.

For all boxplots, the center line represents the median value, box limits represent upper and lower quartiles, and whiskers represent the 1.5× interquartile range. Any points outside of these ranges represent outliers.

## Supplementary information

Supplementary Information

Supplementary Data 1

Supplementary Data 2

Supplementary Data 3

Supplementary Data 4

## Data Availability

Information on the CALGB 40603 clinical trial can be found at the ClinicalTrials.gov website (NCT00861705). The CALGB 40603 targeted panel DNA-seq and phenotypic data used in this paper are available via the NCBI database of Genotypes and Phenotypes (dbGaP) (phs003801.v1.p1), along with the CALGB 40603 RNA-seq data (phs001863.v1.p1)^15^. TCGA whole exome DNA-seq and RNA-seq data are available via the NCBI dbGaP (phs00178). TCGA phenotypic data are available via the NCI Genomic Data Commons (GDC) Data Portal (https://portal.gdc.cancer.gov/projects/TCGA-BRCA)^10^. FUSCC RNA-seq data are available via the NCBI Sequence Read Archive (SRA) (SRP157974). FUSCC probe-level OncoScan CNV Assay data are available via the NCBI Gene Expression Omnibus (GEO) (GSE118527). FUSCC mutation data is available via Figshare (10.6084/m9.figshare.19783498.v5)^47^. FUSCC phenotypic data are available in Jiang et al., with overall survival data provided by request to the first author^19^. METABRIC somatic mutation calls and ASCAT copy number segment data are available via GitHub (https://github.com/cclab-brca/mutationalProfiles/tree/master)^48^. METABRIC phenotypic data are available via cBioPortal (https://www.cbioportal.org/study/summary?id=brca_metabric)^48^. METABRIC expression data are available via the European Genome-phenome Archive at the European Bioinformatics Institute (EGAS00000000083)^20^. All other data supporting the findings of this study are available from the corresponding author upon reasonable request.

## Code availability

The underlying code for this study is not publicly available, but it may be made available to researchers upon reasonable request to the corresponding author.
