# Supplementary material for: Prognostic and molecular multi-platform analysis of CALGB 40603 (Alliance) and public triple-negative breast cancer datasets
Source: NPJ Breast Cancer. 2025 Mar 8;11:24. doi: 10.1038/s41523-025-00740-z (PMC11890565; doi:10.1038/s41523-025-00740-z)
Supplement: Supplementary file 1 — Supplementary Information [file 41523_2025_740_MOESM1_ESM.pdf]

Supplementary Figures:

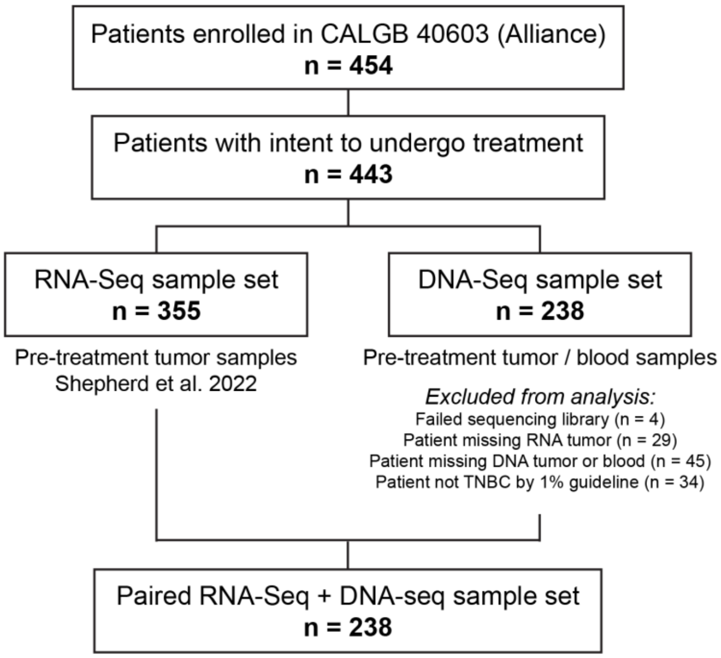

Supplementary Figure 1. Diagram illustrating the sample inclusion criteria for the CALGB 40603 DNA-Seq sample set.

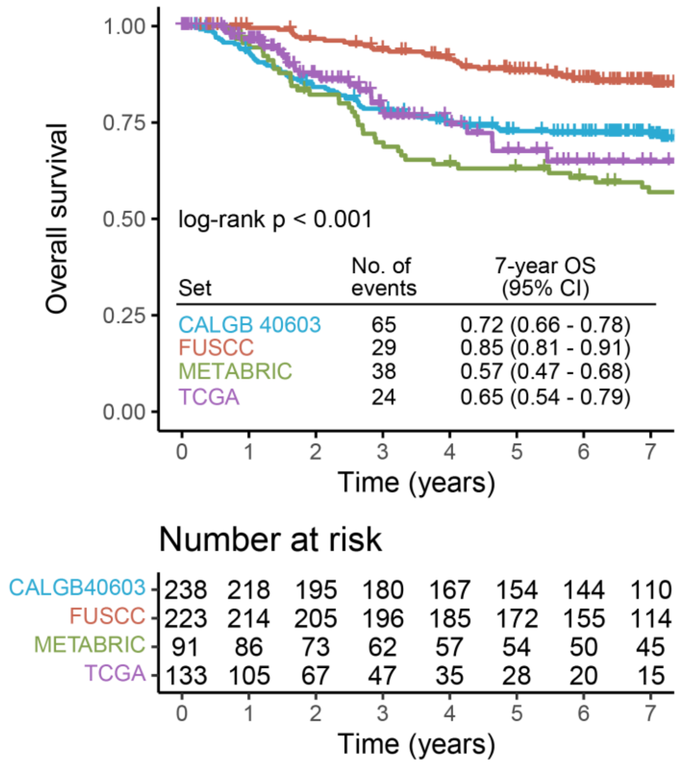

Supplementary Figure 2. Kaplan-Meier plot of overall survival by cohort. CALGB 40603 (blue), FUSCC (red), METABRIC (green), and TCGA (purple) datasets over 7 years. The log-rank test was used to assess the statistical significance of survival differences among the four groups.

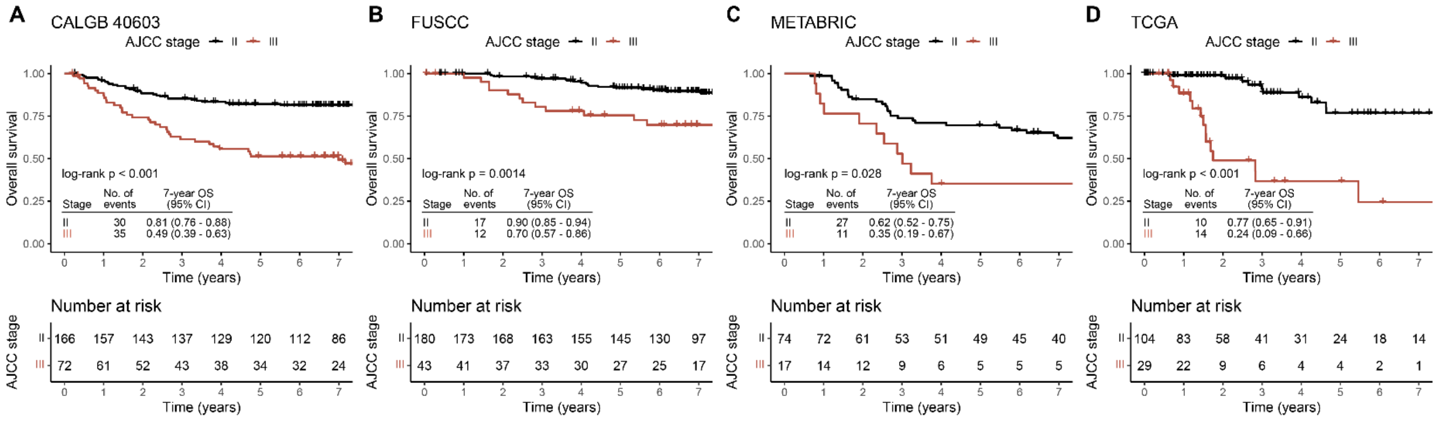

Supplementary Figure 3. Kaplan-Meier plots of overall survival stratified by tumor stage (II-III) in **A,** CALGB 40603, **B,** FUSCC, **C,** METABRIC, and **D,** TCGA. The log-rank test was used to assess the statistical differences between tumor stages.

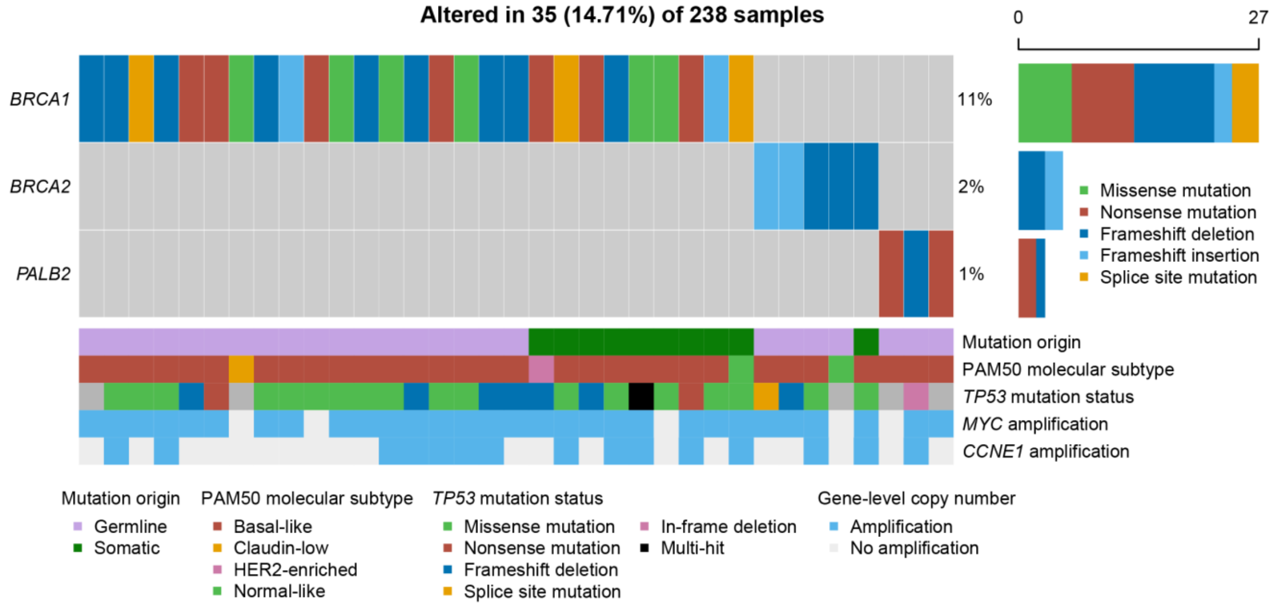

Supplementary Figure 4. Homologous recombination deficiency (HRD) mutations in the CALGB 40603 dataset. The columns correspond to individual patients (n = 35) with a *BRCA1*, *BRCA2*, or *PALB2* mutation (rows). Color-coded labels correspond to mutation type, with light gray representing no pathogenic/oncogenic mutation in that sample. The mutation origin (germline pathogenic/likely pathogenic or somatic oncogenic/likely oncogenic) is annotated at the bottom for each sample, as well as the PAM50 molecular subtype, *TP53* mutation status, *MYC* amplification status, and *CCNE1* amplification status.

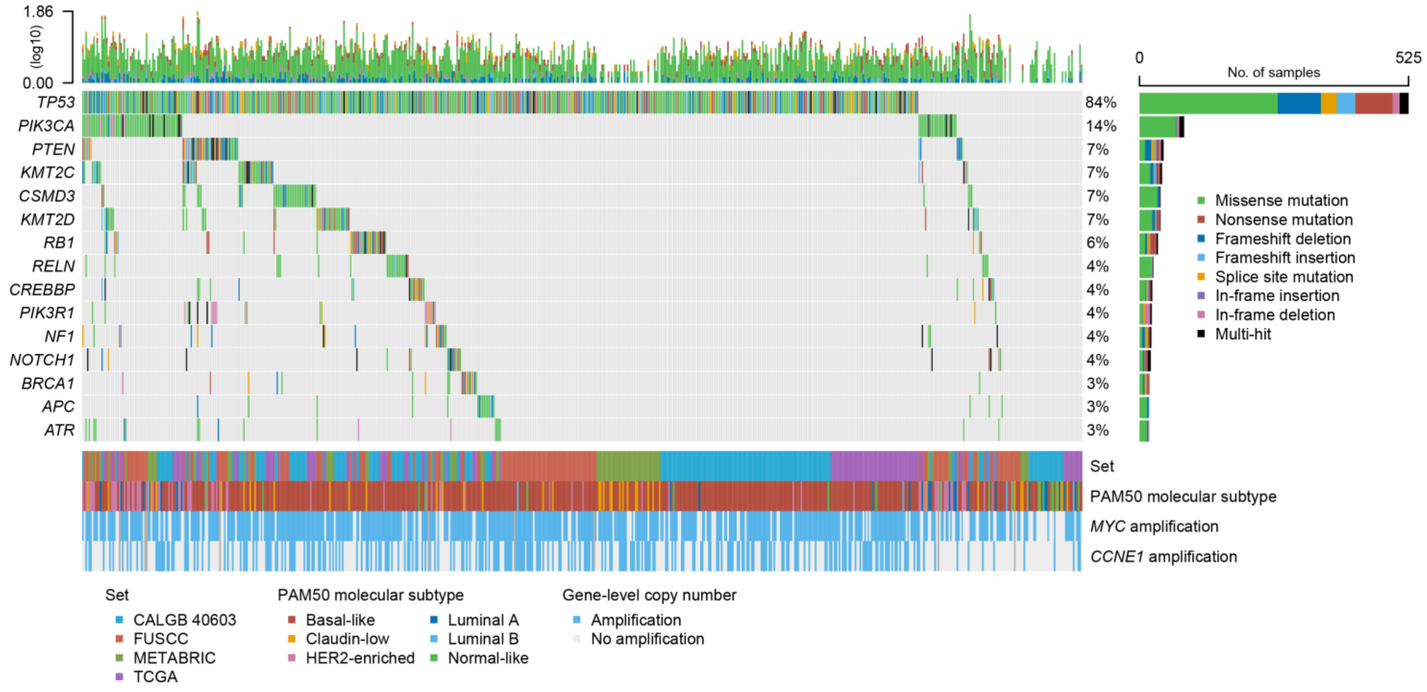

Supplementary Figure 5. The mutational landscape of TNBC samples from four datasets (CALGB 40603, FUSCC, METABRIC, and TCGA). The columns correspond to individual patients (n = 628) and the rows represent mutated features, consisting of the 15 genes with the highest somatic mutation frequencies. Color-coded labels correspond to mutation type, with light gray representing wildtype. Patient-level and gene-level mutation frequency distributions are shown at the top and right, respectively. RNA-based (PAM50 subtype), DNA-based (*MYC* and *CCNE1* amplification) annotations, and dataset annotations are included at the bottom of the plot with corresponding legends.

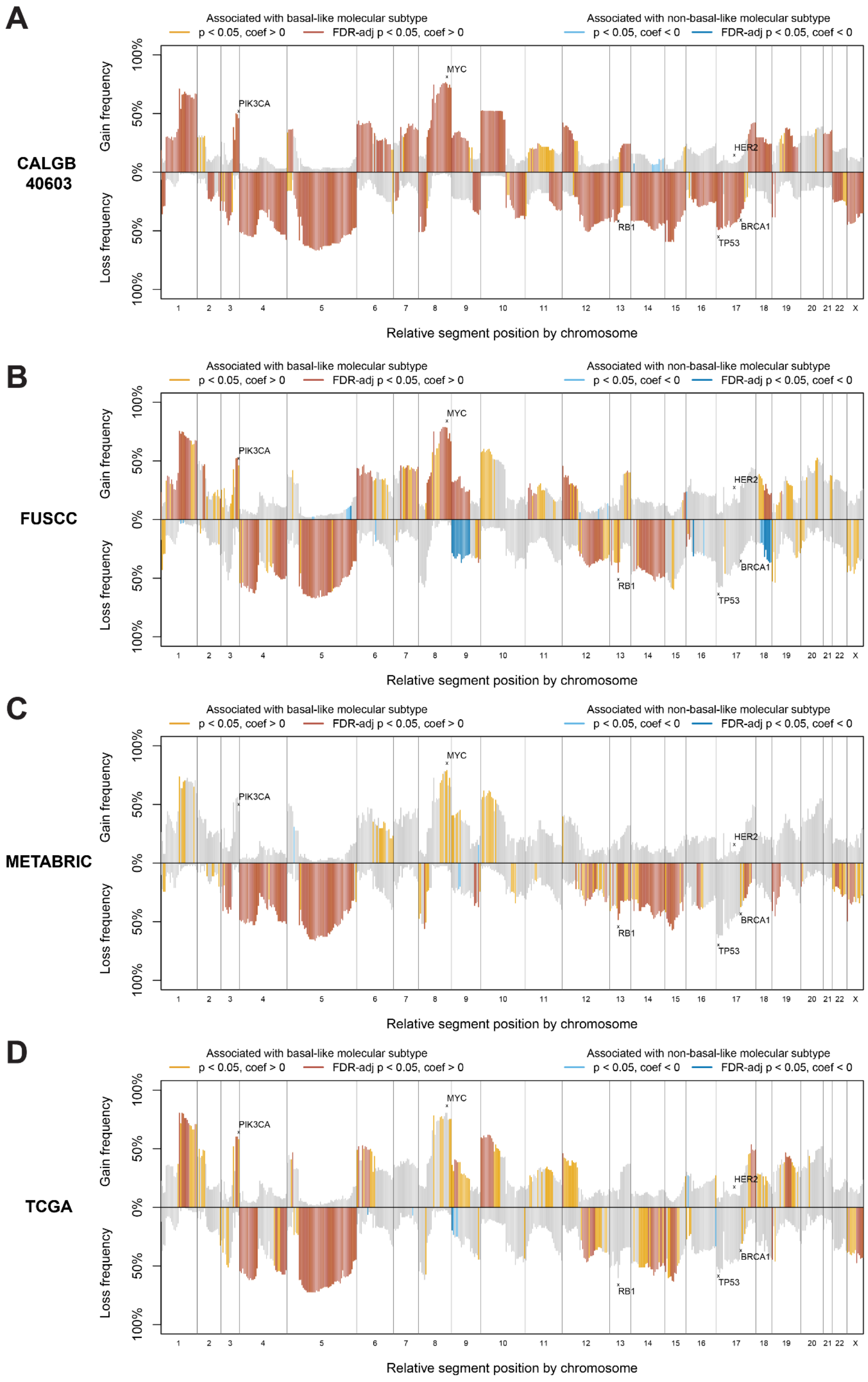

Supplementary Figure 6. Segment-level copy number landscape plots of the **a** CALGB 40603, **b** FUSCC, **c** METABRIC, and **d** TCGA datasets. On the x-axis, each of the 534 copy number segments is plotted in relative order, with height above the x-axis corresponding to the gain frequency of the segment within the sample set and height below the x-axis corresponding to the loss frequency of the segment within the sample set. Segment gain/loss frequencies are colored by statistical significance and direction of association of binomial generalized linear models using segment gain/loss status to predict basal-like subtype. Orange-colored segment gains/losses are statistically more significant in basal-like samples vs. non-basal-like samples, with (dark orange) and without (light orange) multiple test corrections. Blue-colored segment gains/losses are statistically more significant in non-basal-like samples vs. basal-like samples, with (dark blue) and without (light blue) multiple test corrections.

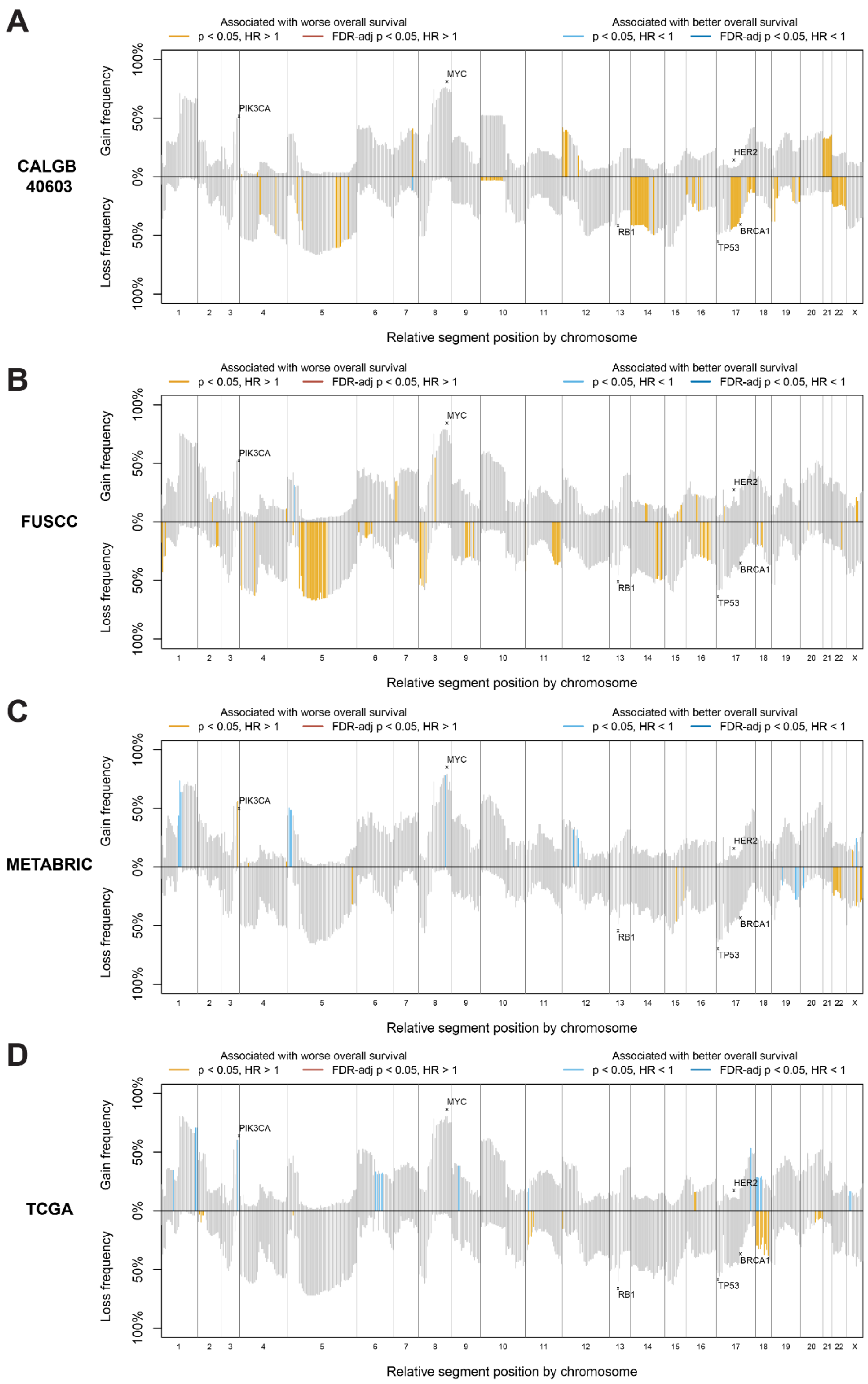

Supplementary Figure 7. Segment-level copy number landscape plots of the **a** CALGB 40603, **b** FUSCC, **c** METABRIC, and **d** TCGA datasets. On the x-axis, each of the 534 copy number segments is plotted in relative order, with height above the x-axis corresponding to the gain frequency of the segment within the sample set and height below the x-axis corresponding to the loss frequency of the segment within the sample set. Segment gain/loss frequencies are colored by statistical significance and direction of association of Cox proportional hazards models using segment gain/loss status to predict overall survival. Orange-colored segment gains/losses are associated with worse survival, with (dark orange) and without (light orange) multiple test corrections. Blue-colored segment gains/losses are associated with better survival, with (dark blue) and without (light blue) multiple test corrections.

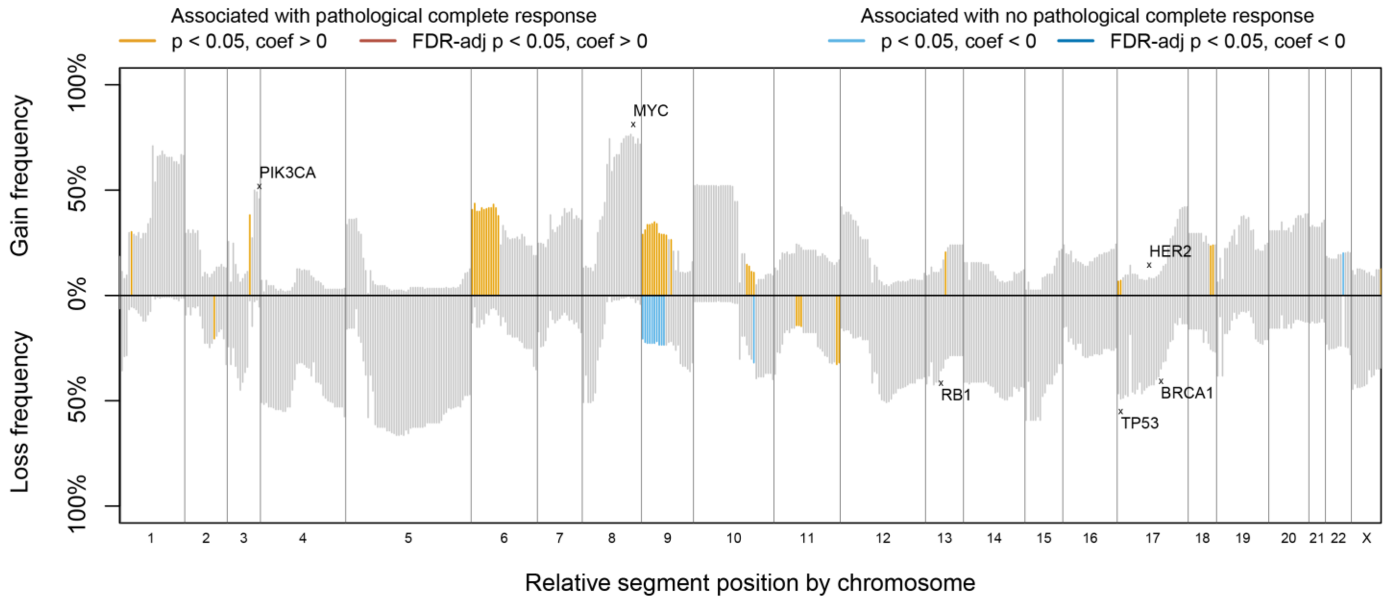

Supplementary Figure 8. Segment-level copy number landscape plot of the CALGB 40603 dataset. On the x-axis, each of the 534 copy number segments is plotted in relative order, with height above the x-axis corresponding to the gain frequency of the segment within the sample set and height below the x-axis corresponding to the loss frequency of the segment within the sample set. Segment gain/loss frequencies are colored by statistical significance and direction of association of binomial generalized linear models using segment gain/loss status to predict pCR status. Orange-colored segment gains/losses are statistically more significant in samples with pCR vs. samples without pCR, with (dark orange) and without (light orange) multiple test corrections. Blue-colored segment gains/losses are statistically more significant in samples without pCR vs. samples, with pCR with (dark blue) and without (light blue) multiple test corrections.

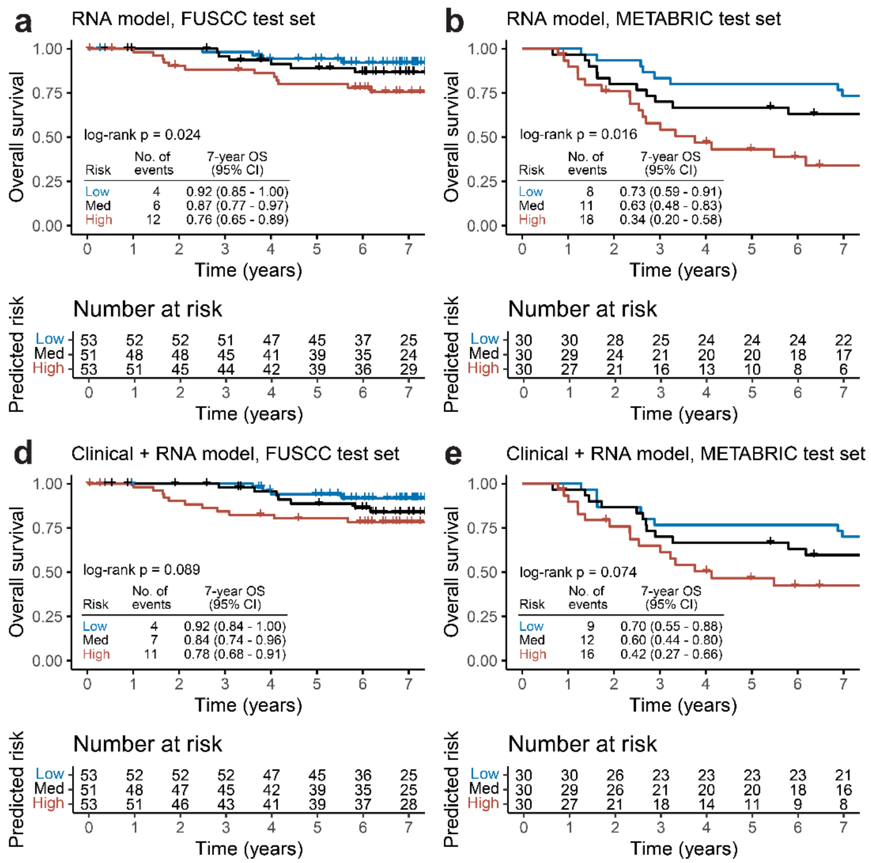

Supplementary Figure 9. Kaplan-Meier plots of overall survival by predicted survival risk from the RNA-only elastic net model in the **a** FUSCC, **b** METABRIC, **c** TCGA individual test sets and from the clinical + RNA model in the **d** FUSCC, **e** METABRIC, and **f** TCGA individual test sets. Continuous risk scores predicted for each sample were categorized into low-risk (blue), medium-risk (black), and high-risk (red) cutoffs based on the median risk score of each test set.

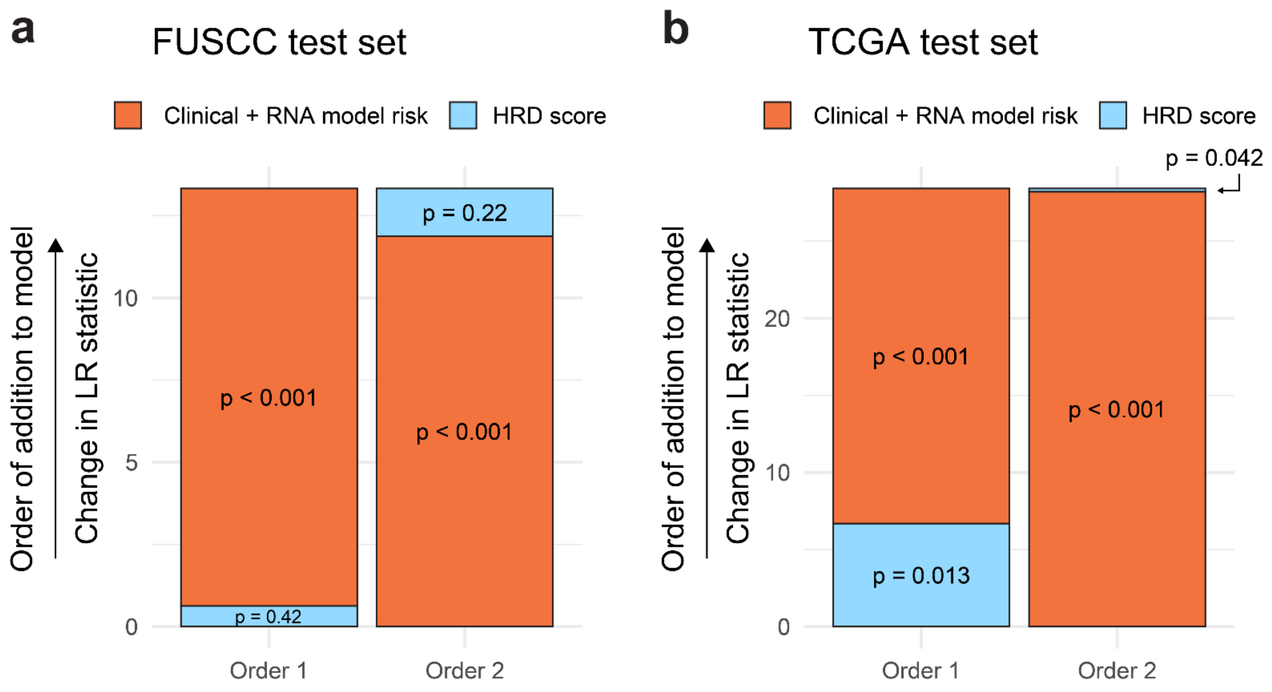

Supplementary Figure 10. The likelihood-ratio (LR) statistic was estimated as we added the continuous clinical + RNA elastic net risk score and/or HRD score to a Cox proportional hazards model using the samples from the **a** FUSCC and **b** TCGA test sets. The change in LR statistic when the HRD score, then the model risk score is added is shown (order 1) alongside the change in LR statistic when the model risk score, then the HRD score is added is shown (order 2). The p-values displayed represent the statistical significance of the corresponding coefficient in the univariate/multivariate model on test set data.

Supplementary Data Legends:

Supplementary Data 1. Results of statistical analyses testing associations of segment-level copy number segments with subtype and overall survival in the four combined datasets, as well as with pCR in CALGB 40603.

Supplementary Data 2. Complete list of 804 published RNA expression signatures and their corresponding references.

Supplementary Data 3. List of selected elastic net model features and coefficients.

Supplementary Data 4. Gene set enrichment results for the three unsupervised RNA signatures selected by elastic net models (Green7, Red18, and Unknown8).
